# Evaluation of Vulnerabilities for the Spread of Carbapenem Resistant Organisms at Five Hospitals in India

**DOI:** 10.1017/ash.2024.252

**Published:** 2024-09-16

**Authors:** Susan Fallon, Umang Agrawal, Shaoli Basu, Valeria Fabre, Sarah Fisseha, Johns Hopkins Hospital, Arjunlal Kakrani, Rajesh Karyakarte, Mahadevan Kumar, Abhijeet Mane, Vidya Mave, Yatin Mehta, SHAHZAD MIRZA, Akaash Patel, Bharat Randive, Prachala Rathod, Matthew Robinson, Camilla Rodrigues, Smita Sarma, Jignesh Shah, Patricia Simner, SWEETY SINGH, Melanie Curless

**Affiliations:** Johns Hopkins Hospital; PD Hinduja Hospital; P.D. Hinduja Hospital and MRC; Johns Hopkins University School of Medicine; Dr D Y Patil Medical College, Hospital & Research Centre; BJ Government Medical College and Sassoon Hospital, Pune; Bharati Vidyapeeth Medical College; Bharati Vidyapeeth (DTU) Medical College and Hospital; Medicine, Johns Hopkins University; Medanta The Medicity; Dr D Y Patil Medical College Hospital And Research Centre, Dr D Y Patil Vidyapeeth, Pimpri, Pune; Byramjee Jeejeebhoy Government Medical College-Johns Hopkins University Clinical Research Site, Pune, India; BJGMC and Sasoon Hospital Pune; Johns Hopkins School of Medicine; Hinduja Hospital, Mumbai; Medanta - The Medicity; Bharati Vidyapeeth University Medical College Pune; Johns Hopkins University

## Abstract

**Background:** The 2022 WHO global survey on infection prevention and control (IPC) exposes significant gaps in IPC in the WHO Southeast Asia region. A better understanding of IPC vulnerabilities will inform improvement initiatives. We describe an evaluation of IPC practices known to prevent and contain carbapenem-resistant organisms (CROs) at hospitals participating in the United States Centers for Disease Control Global Action in Healthcare Network –Antimicrobial Resistance in India. Prior hospital evaluations suggest resistance to carbapenems among gram-negative isolates is up to 45%. **Methods:** We conducted a mixed methods evaluation including cross-sectional surveys, semi-structured interviews, and site observations at five hospitals (one government, two private tertiary care, and two private teaching) located in three cities. The number of hospital beds ranged from 362 to 2,011. Hospital and IPC program characteristics, and CRO prevention and containment activities were examined virtually. Site observations focused on hand hygiene, environmental cleaning, personal protective equipment (PPE), CRO containment practices and use of water for patient care. **Results:** All sites had IPC programs with established policies and qualified IPC staff. The IPC nurse-to-bed ratio ranged from 1:73 to 1:432 (mean, 1:209). Due to the integral role of microbiology staff in IPC at these hospitals, the two departments had strong communication channels associated with CRO identification. Screening for CRO colonization, if done, targeted patients from outside hospitals. Three of the five hospitals routinely implemented contact precautions for patients with identified CROs, displayed isolation signage at the bedside, and provided adequate PPE at point-of-use; however, all sites reported barriers to effective isolation and/or cohorting patients with CROs. Timely communication of CROs to clinical staff varied and no sites effectively relayed CRO status upon patient discharge to another facility. IPC teams identified gaps in environmental cleaning procedures and practices related to medical devices and equipment. All sites used alternatives to tap water for clinical care and sink etiquette was evident. Each IPC team performed audits of patient isolation and hand hygiene practices. Despite the considerable proportion of IPC resources dedicated to daily education and feedback in clinical areas, the IPC teams reported that improvement was often difficult to achieve. **Conclusion:** Given the high burden of CROs and limited IPC resources, detailed knowledge of IPC opportunities for improvement will help hospitals target novel interventions for CRO prevention and containment. Further investigation of colonization rates and effective performance improvement methods in these settings is needed.

**Disclosure:** Patricia Simner: Research Contracts: BD Diagnostics, OpGen Inc., Qiagen Sciences Inc, T2 Diagnostics, Accelerate Diagnostics; Research Collaborators:Ares Genetics, CosmosID, IDbyDNA, Illumina; Consulting: OpGen Inc., BD Diagnostics, Shionogi Inc., GeneCapture, Qiagen Sciences Inc, Entasis, Day Zero Diagnostics, Next Gen Diagnostics

